# Supplementary material for: High Nutritional Conditions Influence Feeding Plasticity in Pristionchus pacificus and Render Worms Non‐Predatory
Source: J Exp Zool B Mol Dev Evol. 2025 Jan 16;344(2):94–111. doi: 10.1002/jez.b.23284 (PMC11788882; doi:10.1002/jez.b.23284)
Supplement: Supplementary file 1 — Supporting information. [file JEZ-344-94-s004.pdf]

Supplementary Table S1. List of crRNA and primer sequences for genes that were studied for mutant analyses.

Gene name	Accession	crRNA	Forward Primer	Reverse Primer
Ppa-pddl-1	ppa_stranded_DN27845_c0_g3_i3	5′-CAGACATTACTATCCTCTAG-3′	5′-CCGTTAGAGTCTACTTCATGCTATGGAA-3′	5′-ATCAACCTGACCATATTTCAGTCTGACC-3′
Ppa-pddl-3	PPA40514	5′-CTATCTCCCCCTTGCGACTC-3′	5′-TTCTGATCTGTGGAACGACCCG-3′	5′-CACGGATTCGACGGGAGTGATG-3′
Ppa-pddl-4	ppa_stranded_DN27845_c0_g2_i1	5′-GTAATTCCCGTCTATTTCTG-3′	5′-TAACGATGTTTTCCTTCAGGTAATGGGC-3′	5′-CTGCTGCTTGTAGATTAGTCCATACAGA-3′
Ppa-dhs-28.1	PPA20393	5′-GGGGAGATCAAGGCAGCCGG-3′	5′-CGATATTGTTGCAGTGAACGAC-3′	5′-CTTCTAGTTACATCAGCTGTCTCG-3′
