# Supplementary material for: High Nutritional Conditions Influence Feeding Plasticity in Pristionchus pacificus and Render Worms Non‐Predatory
Source: J Exp Zool B Mol Dev Evol. 2025 Jan 16;344(2):94–111. doi: 10.1002/jez.b.23284 (PMC11788882; doi:10.1002/jez.b.23284)
Supplement: Supplementary file 2 — Supporting information. [file JEZ-344-94-s003.pdf]

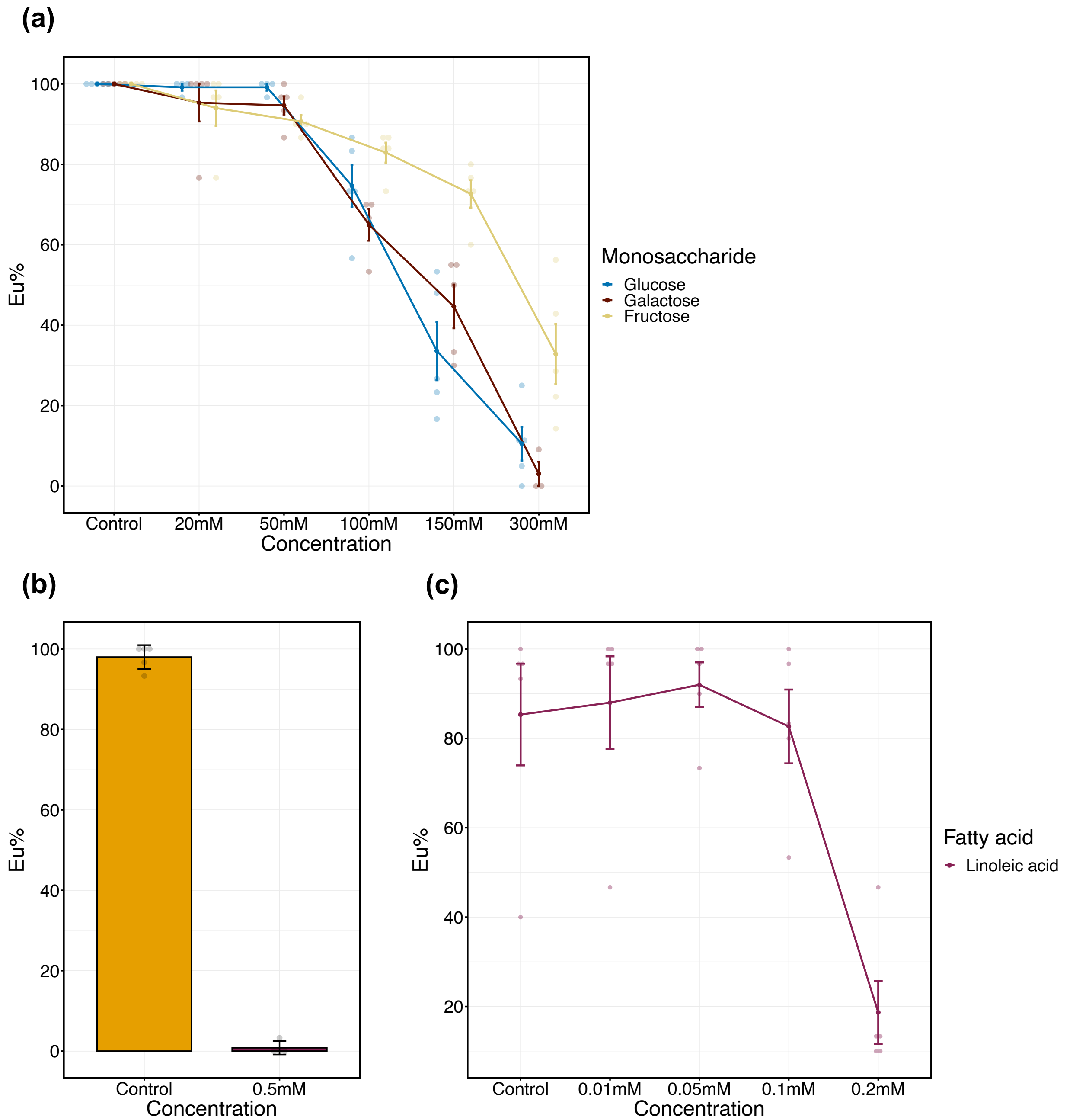

Supplementary Figure S1

Concentration dependent effect of supplements on mouth-form plasticity. (a) Eu percentages of worms grown on different concentrations of monosaccharides. In this pilot experiment, cultures were initiated by inoculating with 10 adult worms, allowing them to lay eggs for 2 hours. This method results in higher Eu percentages than inoculating with 3 worms. N ≥ 3 biological replicates per condition in each concentration. From each replicate (plate), 25-30 worms were scored, except for 300mM concentrations from which 4-28 worms were scored. Error bars represent s.e.m. (b) Effect of 0.5mM linoleic acid on mouth-form plasticity. N ≥ 4 biological replicates per concentration. From each replicate (plate), 28-30 worms were scored. Bars represent mean values of all replicates. Error bars represent s.d. (c) Linoleic acid concentration effect on mouth-form plasticity. N = 5 biological replicates per concentration. From each replicate (plate), 30 worms were scored. Error bars represent s.e.m. (a-c) Each faint data point represents a biological replicate (plate) scored for mouth-form ratio (Eu%).
