# Supplementary material for: High Nutritional Conditions Influence Feeding Plasticity in Pristionchus pacificus and Render Worms Non‐Predatory
Source: J Exp Zool B Mol Dev Evol. 2025 Jan 16;344(2):94–111. doi: 10.1002/jez.b.23284 (PMC11788882; doi:10.1002/jez.b.23284)
Supplement: Supplementary file 3 — Supporting information. [file JEZ-344-94-s001.pdf]

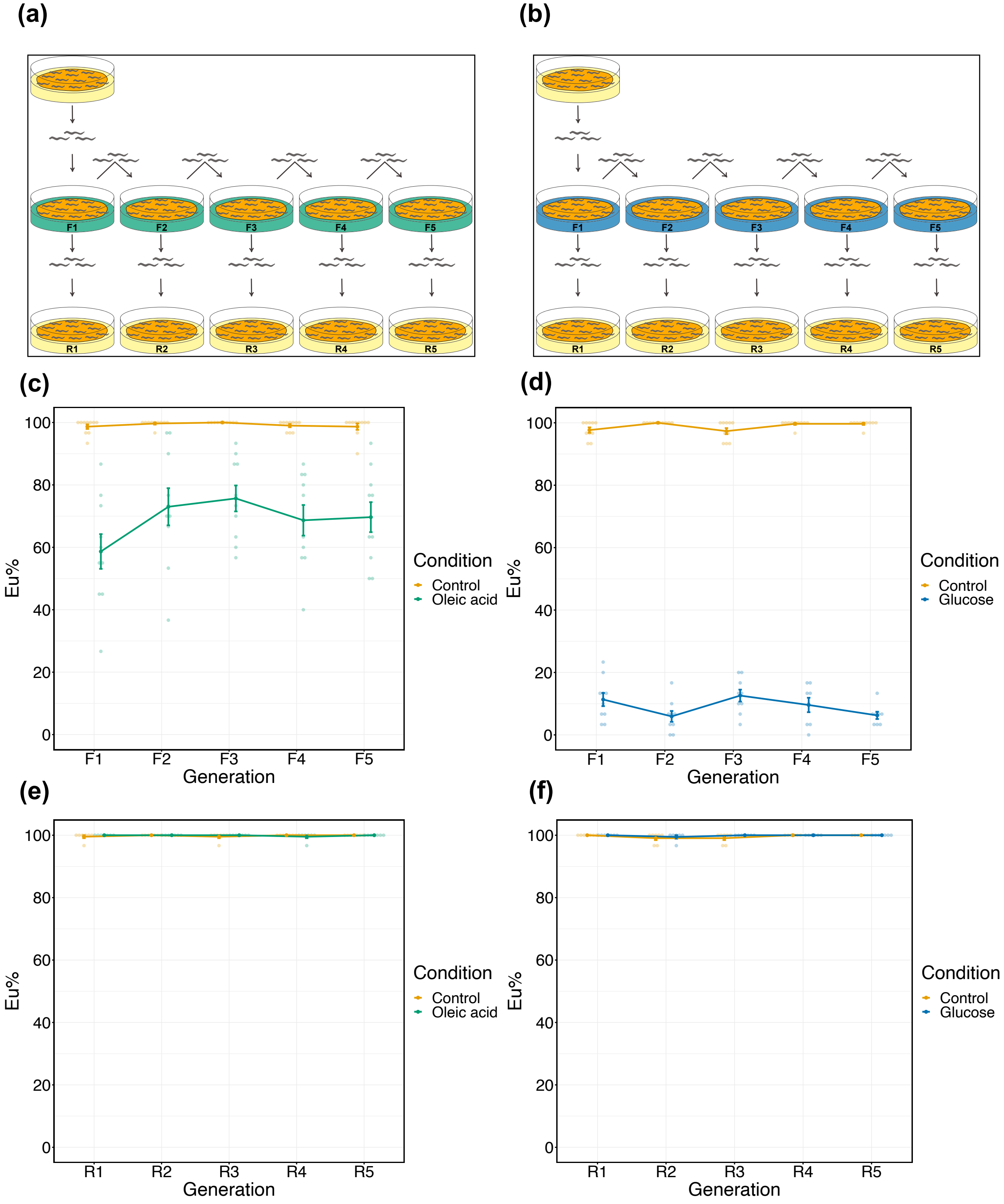

Supplementary Figure S2

Transgenerational effect of oleic acid and glucose supplementations on mouth-form plasticity. (a,b) Illustrations show the experimental design for studying the transgenerational effect of oleic acid (a) and glucose (b) supplementation on mouth-form plasticity. (a,b) The same experimental method was applied for respective control conditions. (c,d) Eu percentages of worms through generations (F1-F5) for oleic acid and control conditions (c); and for glucose and control conditions (d). (c) N = 10 biological replicates per condition for each generation. (d) N ≥ 8 biological replicates per condition for each generation. (e,f) Eu percentage of worms after reversal (F1-F5) for oleic acid and control conditions (e); and for glucose and control conditions (f). (e) N = 7 biological replicates per condition for each generation. (f) N ≥ 6 biological replicates per condition for each generation. (c-f) Each faint data point represents a replicate (plate), with 30 animals per plate being scored for mouth-form percentage (Eu%). Error bars represent s.e.m.
