# Supplementary material for: High Nutritional Conditions Influence Feeding Plasticity in Pristionchus pacificus and Render Worms Non‐Predatory
Source: J Exp Zool B Mol Dev Evol. 2025 Jan 16;344(2):94–111. doi: 10.1002/jez.b.23284 (PMC11788882; doi:10.1002/jez.b.23284)
Supplement: Supplementary file 4 — Supporting information. [file JEZ-344-94-s006.pdf]

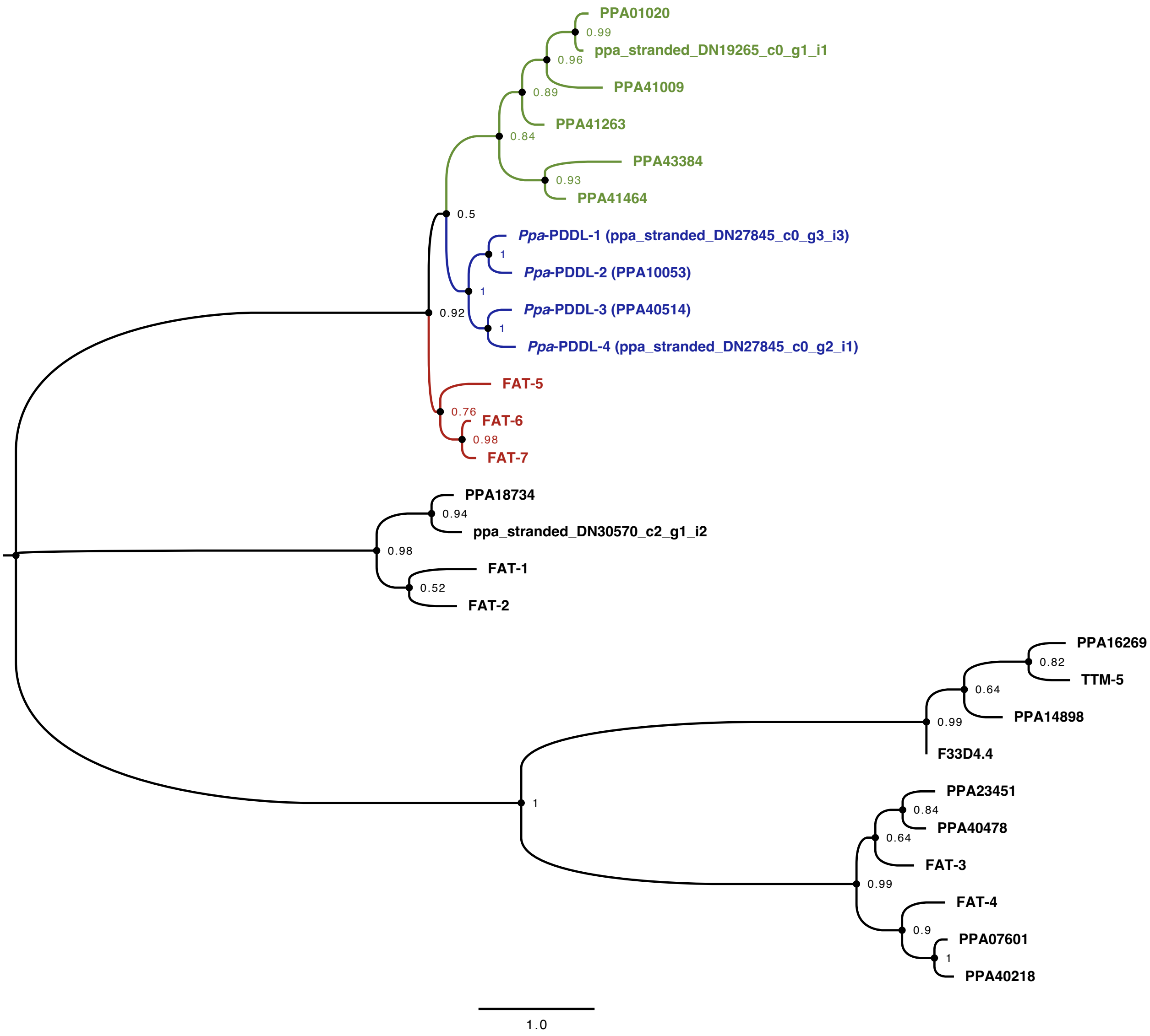

Supplementary Figure S3

Phylogenetic tree of all *P. pacificus* fatty acid desaturase domain-containing proteins with related *C. elegans* desaturases. A maximum likelihood phylogenetic tree, constructed with LG model, and 100 bootstrap replications. Bootstrap values are indicated next to branch nodes. Green and Blue colours denote *Pristionchus* delta-9 desaturase domain-containing proteins. Red colour denotes *C. elegans* delta-9 desaturases.
