# Supplementary material for: High Nutritional Conditions Influence Feeding Plasticity in Pristionchus pacificus and Render Worms Non‐Predatory
Source: J Exp Zool B Mol Dev Evol. 2025 Jan 16;344(2):94–111. doi: 10.1002/jez.b.23284 (PMC11788882; doi:10.1002/jez.b.23284)
Supplement: Supplementary file 5 — Supporting information. [file JEZ-344-94-s002.pdf]

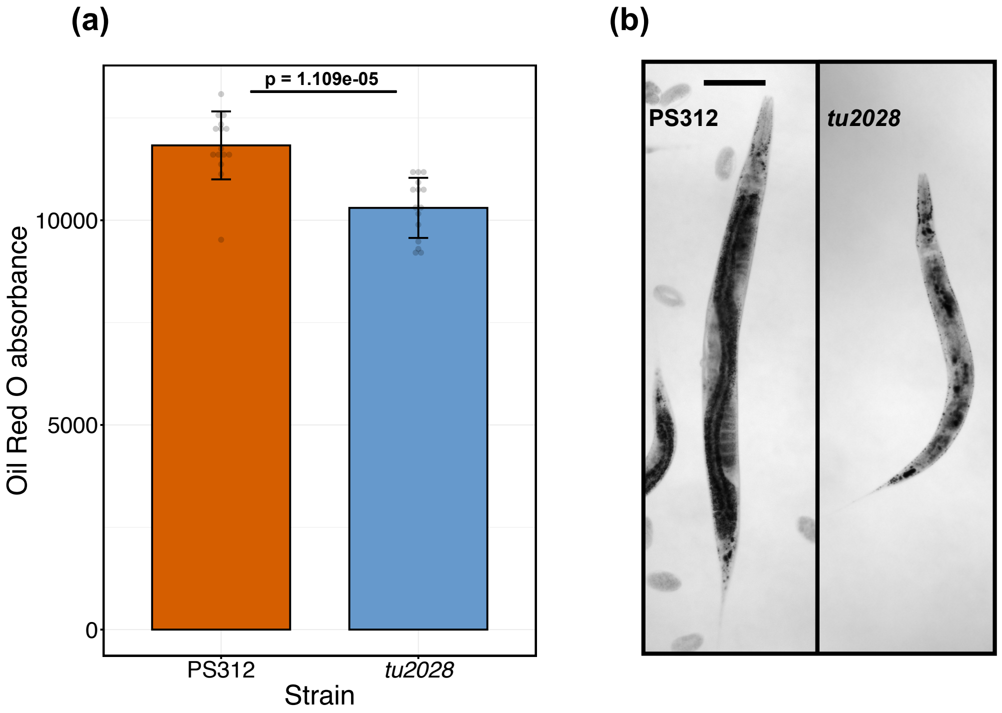

Supplementary Figure S4

Delta-9 desaturase mutant, *Ppa-pddl-1(tu2028)*, exhibits reduced lipid storage relative to wild type strain (PS312) on standard dietary condition. (a) ORO absorbance (RawIntDen/body area) obtained from wildtype (PS312) and *Ppa-pddl-1(tu2028)* strains. N = 15 per strain. P value is obtained from a two sample t-test. Each faint data point represents a worm. Bars represent mean values of all samples for each strain. Error bars represent s.d. (b) Representative images of ORO-quantified worms, indicating lipid storage profile. Images are acquired from the blue channel in grayscale. Lipid droplets appear dark. Scale bar is 100μm.
