# Supplementary material for: High Nutritional Conditions Influence Feeding Plasticity in Pristionchus pacificus and Render Worms Non‐Predatory
Source: J Exp Zool B Mol Dev Evol. 2025 Jan 16;344(2):94–111. doi: 10.1002/jez.b.23284 (PMC11788882; doi:10.1002/jez.b.23284)
Supplement: Supplementary file 6 — Supporting information. [file JEZ-344-94-s005.pdf]

Supplementary Table S2. List of CRISPR mutants utilised in this study.

Gene Name	Gene Accession	Strain	Allele	Mutation Type	Mutation Location	Source
Ppa-pddl-1	ppa_stranded_DN27845_c0_g3_i3	RS4411	*tu2028*	7bp insertion	exon 4	This paper
Ppa-pddl-1	ppa_stranded_DN27845_c0_g3_i3	RS4412	*tu2029*	3bp insertion	exon 4	This paper
Ppa-pddl-3	PPA40514	RS4401	*tu2033*	5bp deletion	exon 5	This paper
Ppa-pddl-3	PPA40514	RS4402	*tu2034*	7bp deletion	exon 5	This paper
Ppa-pddl-3	PPA40514	RS4403	*tu2035*	7bp deletion	exon 5	This paper
Ppa-pddl-4	ppa_stranded_DN27845_c0_g2_i1	RS4398	*tu2030*	4bp deletion	exon 7	This paper
Ppa-pddl-4	ppa_stranded_DN27845_c0_g2_i1	RS4399	*tu2031*	10bp deletion	exon 7	This paper
Ppa-pddl-4	ppa_stranded_DN27845_c0_g2_i1	RS4400	*tu2032*	28bp insertion	exon 7	This paper
Ppa-dhs-28.1	PPA20393	RS4147	*tu1855*	4bp deletion	exon 3	This paper
Ppa-dhs-28.1	PPA20393	RS4138	*tu1856*	7bp deletion	exon 3	This paper
Ppa-dhs-28.1	PPA20393	RS4141	*tu1857*	8bp insertion	exon 3	This paper
Ppa-dhs-28.1	PPA20393	RS4140	*tu1858*	21bp deletion	exon 3	This paper
Ppa-dhs-28.1	PPA20393	RS4139	*tu1859*	19bp insertion	exon 3	This paper
Ppa-daf-22.1;Ppa-daf-22.2	ppa_stranded_DN16812_c0_g1_i1;PPA41516	RS2770	*tu489;tu504*	7bp deletion;7bp insertion	refer to Markov et al., 2016	Markov et al., 2016
sult-1	PPA12547	RS2974	*tu1061*	10bp deletion	exon 9	Namdeo et al., 2018
nag-1;nag-2	PPA06134;PPA34489	RS3195	*tu1142;tu1143*	1 SNP + 17bp insertion;2 SNPs + 9bp deletion	exon 5;exon 5	Sieriebriennikov et al., 2018
